# Homelessness, Politics, and Policy: Predicting Spatial Variation in COVID-19 Cases and Deaths

**DOI:** 10.3390/ijerph20043265

**Published:** 2023-02-13

**Authors:** Hilary Silver, Rebecca Morris

**Affiliations:** 1 Department of Sociology, Columbian College of Arts & Sciences, George Washington University, Washington, DC 20052, USA; 2 Department of Health Policy and Management, Milken Institute School of Public Health, George Washington University, Washington, DC 20052, USA

**Keywords:** homeless, housing, Continuum of Care, COVID-19

## Abstract

When COVID-19 began to spread in the United States, the first public health orders were to hunker down at home. But for the vulnerable people experiencing homelessness, especially those sleeping outdoors, retreating to a private dwelling was not possible. This suggests that places with greater homelessness would also have elevated COVID-19 infections. This paper examines how spatial variation in unsheltered homelessness was related to the cumulative number of cases and deaths from COVID-19. Although Continuums of Care (CoCs) with more households receiving welfare, without internet service, and more disabled residents had a higher rate of COVID-19-related cases and deaths, CoCs with more unsheltered homelessness had fewer COVID-19-related deaths. More research is needed to explain this counterintuitive result, but it may reflect the bicoastal pattern of homelessness which is higher where government intervention, community sentiment, and compliance with rules to promote the common welfare are greater. In fact, local politics and policies mattered. CoCs with more volunteering and a higher share of votes for the 2020 Democratic presidential candidate also had fewer COVID-19 cases and deaths. Yet, other policies did not matter. Having more homeless shelter beds, publicly assisted housing units, residents in group quarters, or greater use of public transportation had no independent associations with pandemic outcomes.

## 1. Introduction

When COVID-19 began to spread in the United States, the first public health orders were to stay at home. But for people experiencing homelessness or sleeping outdoors, retreating to a private dwelling was not possible. Moreover, to remain outdoors with others was to be vulnerable to the virus. It put the unhoused at risk of losing contact with service providers and other assistance, as well as crossing paths with authorities intent on locking down public spaces.

On a single winter night in 2022, roughly 582,500 people were experiencing homelessness in the United States, four in ten of whom were in unsheltered locations such as the street, abandoned buildings, vehicles, parks, or other places not suitable for human habitation [1]. Although there was a slight decline in the number of the homeless in sheltered locations between 2020 and 2022, there was a three percent rise in people experiencing unsheltered homelessness. 

The unsheltered homeless population is distinctive in some ways: being more disconnected from formal employment; having significant physical, mental, and behavioral health challenges; having involvement with the criminal justice system; and experiencing homelessness for longer periods [2]. While 55.4% of all unsheltered people in 2022 were counted in the Continuums of Care (CoCs) that encompass the nation’s 50 largest cities, the rest were split between largely suburban areas (21.0%), largely rural areas (17.6%), and “other largely urban CoCs” (6.0%). Continuums of Care (CoC) are the United States Department of Housing and Urban Development’s geographical jurisdictions, which may cover a city, county, metropolitan area, or an entire sparsely populated state, in which local planning bodies are responsible for coordinating the full range of homelessness services. CoCs as a whole cover virtually the entire area of the United States.

The COVID-19 pandemic posed an unprecedented challenge to the homeless and the people who provide them with services. People experiencing homelessness often stay in congregate shelters, tents, or mobile vehicle communities, making it difficult to avoid contagion. During the initial COVID-19 surge in the winter of 2020, metropolitan counties frequently adopted shelter-in-place orders before their states did [3]. Some shelters imposed restrictions to avoid the risk of exposure. They reduced shelter capacity in order to increase space between people sleeping in congregate settings and to allow for social distancing. In some places, pandemic-related funding allowed for renting hotel rooms and other individual spaces so unhoused people could quarantine and recover from the coronavirus as well as prevent infection in the first place. Yet, from Boise to Vallejo, even some of those relocated to hotels passed away.

For those living on the streets, the Centers for Disease Control (CDC) issued guidelines for outreach workers to provide means of hygiene and disseminate crucial health information. The CDC advised localities not to “sweep” away encampments, but to allow the unsheltered to remain in place, partly so as not to spread disease. Some places, such as Seattle and Santa Clara County, even institutionalized parking lots and campsites where they set up essential services, such as handwashing stations and garbage collection. To prevent even more homelessness, rental assistance was expanded, and eviction moratoriums of varying lengths were enacted. Thanks to policy interventions, homelessness figures remained essentially flat during the first two years of the pandemic.

As suggested by “Stage of Disease” theory [4], initial cases of a pandemic occur in higher SES locales, but in a second stage, some communities implement public health policies more rapidly and systematically to control risk, giving rise to growing socioeconomic inequalities in disease incidence among localities. At the outset of the coronavirus pandemic, some patterns consistent with this theory were discernable. First, there was considerable variation across places, with an epicenter originally in New York and other densely populated and economically integrated places, but soon radiating throughout the country. Lockdowns and mask mandates followed, which required people to self-isolate, socially distance, and if possible, work from home. Those who could not—including people living and working in congregate quarters—were differentially hurt by the coronavirus. Some groups, like the elderly, people of color, and those with preexisting health conditions, were more likely to be infected, and received special attention from authorities such as outreach and information campaigns, as did the homeless. Many high-risk individuals later received priority for the federally approved Pfizer and Moderna mRNA vaccines.

Nevertheless, as the pandemic wore on, infections spread, and new waves of COVID-19 variants began to break through earlier immunizations. In some contexts, efforts to impose mask mandates, close facilities, vaccinate the population, and other public health measures were resisted and short-lived. The United States was not alone in this. Compliance with protective measures varied across countries, with Australia enforcing very strict rules, while antivaccination campaigns opposing public health measures emerged elsewhere, as in Germany. In some developed countries, large numbers of people, especially persons with certain characteristics, chose not to be vaccinated against COVID-19 specifically, due to their deep political beliefs. Indeed, political support for state intervention and the general welfare was associated with the imposition of stricter and longer public health rules during the pandemic. From the beginning, Democrats were much more likely than Republicans to take the threat of the virus seriously and to support efforts to control it [5].

By Spring of 2022, however, many governments were relaxing regulations. As federal and state emergency regulations were lifted, it became possible to assess the cumulative impacts of the pandemic on different places in the US. This paper examines some of the factors associated with spatial variation in overall COVID-19 case and death rates during that two-year period. It particularly examines the local impact of homelessness on the pandemic’s outcomes, hypothesizing that jurisdictions with more unhoused people, many of whom have very low incomes and are disproportionately in poor health, would have more cases and deaths than elsewhere.

Deaths among the unsheltered surged in many cities during the pandemic, often due to chronic conditions that went untreated for long periods [6]. It had become more difficult to go to an emergency room, housing costs rose, and public health authorities were busy combating the coronavirus. One study in San Francisco found that the number of deaths among people experiencing homelessness in the first year of the COVID-19 pandemic were more than doubled since at least 2016. Yet, COVID-19 was not listed as the primary cause of these deaths. Rather, most of the deceased died of acute drug toxicity, especially fentanyl, followed by traumatic injury. This may be because fewer decedents had contact with health or substance use services in the year prior to their death during the pandemic than they did in prior years [7]. Similarly, in New York, there was a rise in deaths among the homeless, some due to COVID-19, but many due to drug overdoses even when housed in hotels [8]. Despite this evidence of high mortality, there has been very little research on the spatial relationship of homelessness and COVID-19 rates.

Studies into the factors responsible for spatial variation in the severity of COVID-19 cases and deaths in the US have mostly examined 3142 counties during the earlier course of the pandemic. The demographics of COVID-19 have received much attention, given the persistent social determinants of many aspects of health. In line with “fundamental cause theory”, socioeconomic (SES) inequalities should have played a cumulative role as the virus spread, producing systematic spatial and individual variation in infections and death [9]. Insofar as the homeless population is disproportionately poor and African American [1], one might expect more adverse COVID-19 outcomes in places with more unhoused residents.

Early research reported higher COVID-19 cases in counties with higher poverty rates and a relatively larger African American population [10,11,12,13]. Authorities expressed concern that information about the virus and then the vaccine was not reaching minority communities. Even if these communities were well-informed, mistrust of medical authorities may have inhibited protective action. The necessity to perform essential work may also have increased the risk of exposure. COVID-19 infections, net of other risk factors, were also higher in counties with greater residential segregation between whites and non-whites [13]. In counties where Black and Latino residents lived in more isolated neighborhoods, especially if the county had a high proportion of front-line workers, these individuals were much more likely to contract COVID-19. Likewise, racial segregation increased COVID-19 death rates for Black, Latino, and white residents [14]. However, as white rates increased over time, racial disparities in infections and death declined [15].

In addition to poverty and racial composition, the extent of COVID-19 cases and deaths in the US varied with lower educational levels and population or housing density [5,11,12]. Counties with more unemployment, multiunit households, nursing home residents, households without a vehicle, and limited English language proficiency were also disproportionately impacted [12]. Distance to major international airports and the share of elderly individuals in a locality also increased risks of COVID-19 early in the pandemic, but by late 2020, these associations disappeared [11].

Under the Trump Administration, policy responses and information campaigns were largely left to the states and localities. One might expect that jurisdictions with more resources would be able to acquire more Personal Protective Equipment, masks, testing kits, vaccines, and other means to protect their residents. Yet, research into the influence of health-related policies on COVID-19 outcomes is just beginning. One inventory of the initial wave of almost two dozen measures of containment, closure, economic support, and public health policies was made in early 2021 for a sample of 171 counties in 7 states [16]. While policies tended to be related, there was significant variation found within and across states in their number and comprehensiveness. With respect to closures, public transportation was most frequently affected. About three-fourths of counties offered housing support and coordinated public information campaigns, while two-thirds required masks outside the home. Another study found that shelter-in-place orders not only increased distancing, especially in urbanized and densely populated counties, but also that urban counties that adopted the order early on had substantial reductions in COVID-19 case growth two-and-a-half weeks later [17].

State public health policies in 2020 were stricter in states with Democratic rather than Republican governors [18]. Moreover, 86% of Democrats reported wearing a face mask every time they left their homes and might be in contact with others, compared to 48% of Republicans, according to a July 2020 poll by NBC News Survey Monkey. Partisanship, rather than geography or severity of COVID, influenced state mitigation policies, at least through the rise of the Delta variant [19]. The politicization of public health measures to stem the spread of the coronavirus was related to the impact of the pandemic across the country. In the first few months, COVID-19 testing, test positivity, incidence, and death rates were lower in US states with Republican governors, but in June to July 2020, the relationship reversed, so that overall, from 15 March 2020 through 15 December 2020, COVID outcomes were positively related to the Republican Party affiliation of governors across the 50 U.S. states and the District of Columbia [20].

Others who tracked the virus at the county level over longer periods also found that COVID-19 mortality through 31 October 2021 was related to the partisan divide [21]. Trump-leaning counties were less severely affected early on, but by 1 March 2021, when vaccines were readily available, this situation had reversed, and in counties with a high percentage of Republican voters, vaccination rates were significantly lower and COVID-19 cases and deaths per 100,000 residents were much higher [5,11]. COVID-19 vaccine uptake, as reported by the CDC, explained approximately 10% of the difference, suggesting that voting preferences are a proxy for compliance with and support of public health measures that would protect residents from COVID-19.

Similar findings were reported for cross-sectional COVID-19 mortality rates and stress on hospital intensive care unit capacity in the 435 US Congressional Districts between April 2021 and March 2022 after vaccines were available to adults. These adverse COVID-19 outcomes were higher in conservative places, as reflected in the District’s US Representative’s and Senator’s concurrent overall voting record and in their specific votes on COVID-19-related matters. Such voting also reflected the extent to which the state’s Governor, State House, and State Senate were all under Republican control. Related to this, liberal counties in conservative states were more than twice as likely to adopt a shelter-in-place policy and implement one earlier in the pandemic, possibly because urban local governments are more driven by risk aversion and necessity to step into the void left by state or federal inaction [22]. At the individual level, too, political affiliation with the Republican party was associated with excessive death rates during the COVID-19 pandemic, especially after vaccines were widely available [23]. Compliance with mask rules also tracked partisan patterns. Mask use was high in the Northeast and the West, and lower in the Plains and parts of the South, but with many fine-grained local differences. Masks were widely worn in the District of Columbia, for example, but not so much in sections of the Maryland and Virginia suburbs [24].

In summary, one would expect that in places where voters and their representatives lean toward the Democratic Party, there would be more public health measures to protect against the coronavirus and they would be complied with. These places should also have more extensive affordable housing and antipoverty programs.

Related to this is evidence that the impact of the pandemic varied with local community engagement. One study reported that trust in state government and local health officials was positively associated with protective health behaviors during the pandemic, especially among Republicans, but trust in the federal government was associated with a lower likelihood of such behaviors [25]. People who trust and feel responsible for others in their community are not only likely to follow public health directives such as masking in public, but also to volunteer to help others. Voluntary, compassionate, and unpaid actions for the benefit of someone or others outside the immediate family are supposedly apolitical and lead to many desirable social outcomes [26].

## 2. Materials and Methods

In what follows, we present a negative binomial regression analysis that tests whether spatial variation in homelessness is associated with more cumulative COVID-19 cases and deaths. We also control for many of the political, policy, and demographic factors that prior research found to be associated with these outcomes earlier in the pandemic across states and counties.

The units of analysis for the study are Continuums of Care, which are geographical areas in which local planning bodies coordinate homelessness services. The regressions are based on 374 cases after deleting CoCs in territories like Puerto Rico, those that merged or split over time, or those missing data on some variables. The choice of the CoC was driven by a desire to minimize measurement error in the homelessness statistics. Around 41% (164) of CoCs are single counties in which the boundaries line up perfectly. But for others, we have followed a procedure recommended by Thomas Byrne [27] to match census tracts or counties to CoCs. Around 32% of CoCs are combinations of counties whose territories are confined to the CoC. In 8% of the CoCs, a single county is split between multiple CoCs, so a weighted adjustment is made based upon the relative sizes of the county’s territory in each. In another 18% of the cases, the CoC is a combination of multiple counties as well as part of at least one other county, so weights are used to adjust the partial county data and assign it to a given CoC.

Only about 12% of CoCs are major cities, and another 15% are largely urban. Forty-four percent of the CoCs are largely suburban, while 28% are largely rural. Homelessness is of course more of an urban phenomenon [1], but COVID-19 rates were no higher in major cities than in other CoCs in our sample.

The dependent variables are cumulative COVID-19 case and death rates per 10,000 in the population. The data were collected by *The New York Times* directly from state and county health departments. The cases and deaths were weighted from the county to the CoC level (https://github.com/nytimes/covid-19-data, accessed on 14 September 2022).

The case and death rates were estimated with negative binomial regression analyses, with cluster-robust standard errors to adjust for potential state-level clustering [28,29]. Negative binomial regression is appropriate for estimating the net effect of predictor variables on an over-dispersed count outcome variable, that is, when the mean of the count is lesser than the variance of the count. Initially, the analysis also predicted case and death rates at six-month intervals of the pandemic, but the correlates remained largely the same over time. Thus, this paper presents the cumulative case and death rates as of April 2022, the latest data available at the time of our analyses.

**Figure 1:**
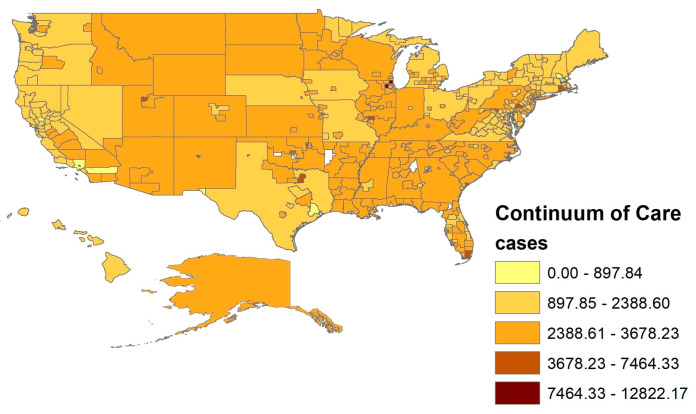
COVID-19 Case Rates by Continuum of Care.

**Figure 2:**
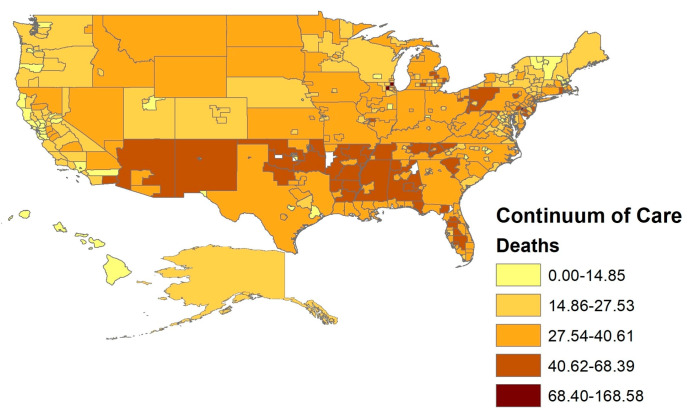
COVID-19 Death Rates by Continuum of Care.

These data were supplemented with seven-day COVID-19 county-level transmission rates published every four months by the Centers for Disease Control (https://data.cdc.gov/Public-Health-Surveillance/United-States-COVID-19-County-Level-of-Community-T/8396-v7yb, accessed on 14 December 2022). The total number of new cases per 100,000 persons within the last seven days was weakly negatively correlated with the cumulative number of COVID-19 cases in the New York Times data. As mentioned, places that initially lagged caught up to those that led the pandemic’s carnage over time. The maps below depict the spatial distribution across CoCs of the cumulative two-year COVID-19 cases and deaths (see Figure 1 and Figure 2).

Table 1 presents the descriptive statistics for the variables in the models. Rates are standardized to population size across the CoCs. The key independent variable is the number of unsheltered homeless people per 100,000 in population in 2020. Total homelessness was also examined. These numbers come from the Point-in-Time counts of CoCs on a single night in late January 2020 and published just prior to the pandemic, which delayed the count in many places during 2020 and 2021. The annual data are published by the Department of Housing and Urban Development (HUD). The rates of people experiencing homelessness who are unsheltered, who are also the most vulnerable to COVID-19 among this already vulnerable group, varied more systematically with COVID-19 case and death rates.

A number of factors related to the unsheltered homelessness rate may confound any apparent correlation between it and COVID-19 rates,. These potentially confounding variables are controlled in the negative binomial models. Many measures of these control variables were extracted from the five-year American Community Survey (ACS) of 2015–2019. These ACS data were the most recent data available at the time of the analysis. Although this may mean the demographic variables are somewhat “outdated” compared to information from the single-year 2019 ACS, it allows for tract level rather than county level estimates, improving the geospatial accuracy of these variables along the borders of CoCs. For data available only at the county level, we relied on weighting the county values by county population relative to total population within the CoC borders.

Among the covariates were median rents and many other housing variables that were correlated with COVID-19 cases and deaths but did not have independent effects in the models with homelessness and other factors controlled. The models contain two housing variables from 2019 that might alleviate unsheltered homelessness. Homeless Beds per Homeless Person refers to HUD data on the number of shelter beds in the CoC divided by the overall PIT count of the homeless population. The number of publicly assisted housing units—i.e., public housing, Section 8 subsidized housing, and so on—per household in the CoC was derived from the National Low Income Housing Coalition’s National Housing Preservation Database as of January 2021, the only deduplicated source for comprehensive data on the publicly supported housing property inventory.

The five-year (2015–2019) ACS estimates of the share of the CoC population living in group quarters, the share using public transportation to go to work, and the share living in households with no internet access were also held constant. Group quarters is a category that includes dense institutionalized living arrangements such as adult correctional facilities and nursing homes as well as college dormitories, crowded conditions that led many authorities to deconcentrate and send the residents home at the pandemic’s height. Traveling to work on crowded public transportation likewise put workers at greater risk of infection. Not having internet during the crisis also made it difficult to get public health information, continue schooling or working remotely, and register for vaccinations.

At the outset of the pandemic, people with preexisting medical conditions, the elderly, and people of color were also exceptionally vulnerable to the virus, so the model includes the population shares of those over 65 years old, those with a disability, and those who self-reported as Black or African American alone in the ACS. Obviously, these categories disproportionately characterize the unsheltered and partially overlap with one another. The shares of the Hispanic and Native populations had no relationship with COVID-19 rates in our data.

Prior research indicates that controlling for socioeconomic conditions in ecological models is a complex endeavor due to multicollinearity. Analyses of the ACS data prior to finalizing the models presented here found that poverty rates, median income, and share of the population with at least a college degree were all associated with cumulative COVID-19 cases and death rates. However, among this set of interrelated SES indictors, the best single predictor of cumulative COVID-19 cases and deaths was the share of households receiving cash public assistance, including Temporary Assistance for Needy Families (TANF, sometimes called “welfare”, and the Supplemental Nutrition Assistance Program (SNAP, formerly known as Food Stamps). These programs target very low-income households, and their generosity and eligibility rules vary by state. However, the shares of those on federal Supplementary Security Income for the disabled, on Medicare (public health insurance for the elderly), or the share of residents without health insurance at all were unrelated to variation in COVID-19.

Other local health conditions were considered in addition to disability rates, using the county health rankings of the University of Wisconsin Population Health Institute. Of all these, the percentage of fee-for-service Medicare enrollees that had an annual influenza vaccine in 2019 was taken as a good indicator of how receptive the local population would be to COVID-19 vaccination as well.

In light of prior findings, two political variables were also retained in the model. First, an indicator of concern for the public good was selected from Opportunity Insights’ interrelated measures derived from the Facebook data atlas (https://dataforgood.facebook.com/dfg/tools/social-capital-atlas, accessed on 14 December 2022; see [30,31]). More precisely, this is the percentage of Facebook users who are members of a group predicted to be about “volunteering” or “activism” based on group title and other group characteristics. Excluded are groups that have a ‘secret’ privacy status, and noise was added to protect privacy. Second, the average percentage of votes for the Democratic presidential candidate in 2020, based on data reported by the MIT Election Lab, was controlled.

Finally, the average annual temperature in CoC was also held constant because unsheltered homelessness is associated with climate variation [32].

## 3. Results

The negative binomial regression analyses are presented in Table 2. Generally, the variables predicting COVID-19 case rates and death rates are similar. In the model of cases, unsheltered homelessness rates had no effect, although overall homelessness rates did have a significant impact (*p* < 0.04, not shown). In the model of deaths, the rate of unsheltered homelessness is significantly and negatively associated with cumulative COVID-19 outcomes. The unexpected sign of the effect is also present in the bivariate correlations, in a log transformation of the homeless rate, as well as after other factors are controlled. All other covariates held constant; a one-unit increase in the number of unsheltered individuals for every 100,000 people in a CoC is associated with a difference in the logs of expected cumulative deaths per 100,000 people in a CoC of −0.007. In other words, an additional unsheltered individual per 100,000 people is expected to decrease the cumulative rate of COVID-19 deaths by a factor of 0.99. Given that the mean cumulative COVID-19 death rate in this sample is 30.2, we can expect that, at the mean, an additional unsheltered homeless person per 100,000 people is associated with 0.30 fewer COVID-19 deaths per 100,000 people [33].

Regardless of unsheltered homelessness, the share of the CoC population with disabilities is also independently related to both COVID-19 outcomes, but differently. A one-unit increase in the percent of individuals who had a disability is associated with a difference in the logs of expected cumulative COVID-19 cases per 100,000 people in a CoC of −2.52, a relationship significant at the 0.001 level. Therefore, a one-unit increase in the percentage of individuals who are disabled within a CoC is associated with a decrease in the rate of cumulative COVID-19 cases by a factor of 0.08, holding all other factors constant. A one-unit increase in the percent of individuals who have a disability is associated with an increase in the rate of COVID-19 deaths per 100,000 people in a CoC by a factor of 21.76. The difference in signs between cases and deaths could be due to disabled individuals taking more precautions to avoid COVID-19 precisely because they were more likely to die from catching the disease.

Another variable with a strong positive impact on COVID-19 cases and death rates is the share of public assistance recipients. Recall that this variable is strongly associated with other indicators of SES. Socioeconomic vulnerability clearly took its toll during the pandemic. However, age, race, assisted housing, and living in group quarters added little to the explanation once other factors were controlled. One reason for the lack of a race effect may be that, over the course of the pandemic, the gap in age-adjusted COVID-19 death rates between Black and white people narrowed. By the autumn of 2021, with the exception of the peak of the omicron wave, Black rates were actually lower than white rates [15].

By far the strongest predictor of cumulative COVID-19 outcomes was the share of the vote won by the Democratic candidate in 2020. A one-unit increase in the percentage of voters in a CoC that voted for Joseph Biden, the Democratic candidate in the 2020 presidential election, is associated with a decrease in the expected rate of cumulative COVID-19 cases by a factor of 0.56. Similarly, a one-unit increase in the percent of voters in a CoC that voted for the Democratic candidate in the 2020 presidential election is associated with a decrease in the expected rate of cumulative COVID-19 cases by a factor of 0.33. Volunteering also had a significant negative, independent effect on both the COVID-19 case and death rates. Democratic voting and volunteering may imply greater trust in and compliance with stricter local public health measures and better COVID-19 outcomes, consistent with research discussed above. Conversely, other things equal, areas with more households without internet service had a higher rate of COVID-19 cases and deaths, possibly because these households did not have access to public health announcements or needed to venture outside for work or to purchase necessary goods.

Finally, some factors had no significant effects. Areas with more homeless shelter beds, publicly assisted housing units, residents in group quarters, or greater use of public transportation had no independent associations with pandemic outcomes. Race, age, and average annual temperature likewise had no significant effect.

## 4. Discussion

The negative relationship between unsheltered homelessness and COVID-19 rates is perplexing, especially once potentially confounding effects are held constant. Controlling for the average temperature, for example, had no impact, although it is significantly related to unsheltered homelessness. Although the cumulative COVID figures are added up through April 2022, when the weather is milder than in December, we were predicting two years’ worth of cases and deaths. The provision of more shelter beds and publicly assisted housing units, which might be expected to account for unsheltered homelessness rates, did not have an independent effect on COVID-19 cases or mortality. The negative effect of unsheltered homelessness persisted despite controlling for potentially confounding effects of local disability rates, racial and age composition, and broader socioeconomic disadvantage, all characteristics of the unhoused that should increase COVID-19 susceptibility.

The negative association is intriguing and calls for more research. After all, cities such as Los Angeles, Seattle, and New York made special efforts to protect the homeless during the pandemic, such as renting hotel rooms, distributing emergency housing vouchers, street outreach, and in some cases, tolerating tent cities and encampments. The unexpected finding could be because these liberal CoCs adopted measures that not only protected rough sleepers, but the overall population as well. This would conform with a 2021 USICH report that suggests that homeless populations fared better than initially expected as a result of “early and firm action” and that claims COVID-19 deaths among people experiencing homelessness have been “significantly and dramatically lower than had been originally projected” [34].

Indeed, the strong negative effect of the share of the Democratic presidential vote suggests that “Blue” cities and states, Democrat-run jurisdictions associated with liberal policies and government intervention, made good policy decisions in the long run, despite early surges of the virus. Yet, even in Republican jurisdictions, other-regarding attitudes and behavior may have had an independent effect on the spread of the disease.

## 5. Conclusions

This analysis found that CoCs with greater homelessness did not have higher overall COVID-19 cases or deaths. This was contrary to expectation, since other state and county indicators of low SES and vulnerability were associated with the pandemic’s ill effects. This counterintuitive result may reflect the underlying bicoastal pattern of homelessness, which is higher where government intervention, legitimate rules, support for the common welfare, and Democratic partisanship are also greater. Another possibility is that people living on the street actually benefit from the germicidal open-air factor and relatively rare person-to-person COVID-19 transmission outdoors [35,36].

The strongest determinants of COVID-19 outcomes were political and policy variables. Democrat-led governments were more likely to institute public health measures such as mandatory or strongly encouraged shutdowns, masking, testing, and vaccinations that, our findings suggest, were apparently effective in reducing the pandemic’s toll. Understanding the relationship between politics and public health measures can better prepare communities for a future crisis. Unfortunately, polling data show that Americans remain sharply divided by party over COVID-19, with a growing share of Republicans who believe the worst of the pandemic is over [37].

Partisanship aside, the independent protective effects of greater volunteering and vaccination demonstrate that trusting public authorities, altruism, and taking account of others in a crisis can have positive benefits for the community. Places with greater dependence on means-tested programs for the poor, however, were associated with more COVID-19. They have larger low-income populations who were more vulnerable to the virus. The pandemic highlighted a need to strengthen social protection systems to make them more responsive to public health crises.

Several caveats regarding the findings are in order. First, this is an ecological analysis at the level of CoCs, which themselves vary a great deal in size and area. We adjusted for clustering by state, but cannot address individual-level factors that might account for personal COVID-19 outcomes. Nevertheless, CoCs must make policy decisions for their jurisdictions based upon the best available aggregate information. We tried to analyze the most up-to-date and reliable data, but are aware there is potential measurement error in in the COVID-19 case numbers, especially after widespread testing was suspended, and in COVID-19 mortality reporting when comorbidities were present (“dying with, but not from, COVID-19”). Even the government admits that official figures undercount unsheltered homelessness. In summary, more systematic data are needed to assess how coronavirus infections and deaths are related to local homelessness and to determine specific policy measures to address both problems.

## Figures and Tables

**Table 1:** Descriptive Statistics at the Continuum of Care Level.

Variables	Mean	Standard Deviation	Minimum	Maximum
Cumulative COVID Case Rate	2499.89	1173.82	0.18	12,822.17
Cumulative COVID Death Rate	30.20	17.92	0.00	168.58
Percent Voters Democratic	0.44	0.18	0.01	0.89
Unsheltered Homeless Rate	6.87	12.10	0.00	103.13
Homeless Beds per Homeless	1.91	1.04	0.21	8.27
Public Transit Use Rate	0.03	0.06	0.00	0.56
Percent Vaccinated in 2019	0.41	0.11	0.01	0.63
Percent Without Internet	0.14	0.09	0.00	0.56
Volunteering Rate	0.07	0.03	0.00	0.23
Assisted Units per Household	0.10	0.32	0.00	5.37
Percent Black	0.12	0.12	0.00	0.78
Percent Aged 65+	0.16	0.03	0.09	0.31
Percent Disabled	0.13	0.03	0.00	0.22
Percent using TANF/SNAP	0.12	0.05	0.03	0.39
Percent in Group Quarters	0.03	0.02	0.00	0.14
Average Temperature	56.74	8.07	33.40	78.00

**Table 2:** Regression Coefficients (Standard Errors) on Cumulative COVID-19 Cases and Deaths. (as of 30 April 2022).

Variables	Cases per 100 K	Deaths per 100 K
Percent Voters Democratic	−0.58 ***	−1.10 ***
	(0.19)	(0.29)
Percent on TANF/SNAP	1.28 *	1.51 *
	(0.58)	(0.80)
Percent Vaccinated in 2019	−0.005	−0.00
	(0.004)	(0.01)
Percent Without Internet	0.25 *	0.49 **
	(0.15)	(0.25)
Volunteering Rate	−1.46 **	−3.85 ***
	(0.67)	(1.39)
Unsheltered Homeless Rate	−0.003	−0.007 **
	(1.34)	(0.003)
Homeless Beds per Homeless Person	−0.02	0.01
	(0.012)	(0.02)
Assisted Units per Household	3.56	1.42
	(4.10)	(4.19)
Percent Black	0.03	0.15
	(0.25)	(0.31)
Percent Aged 65+	0.35	1.06
	(0.94)	(1.05)
Percent Disabled	−2.52 ***	3.08 ***
	(0.70)	(1.13)
Percent in Group Quarters	−0.35	−1.52
	(1.34)	(1.64)
Average Temperature	−0.004	0.001
	(0.002)	(0.004)
Public Transportation Rate	−0.66	0.80
	(0.71)	(1.06)
Constant	8.72 ***	3.24 ***
	(0.33)	(0.41)
Observations	374	374
Pseudo R-squared	0.0138	0.0711

Standard errors in parentheses. *** *p* < 0.01, ** *p* < 0.05, * *p* < 0.1.

## Data Availability

The data used for this study may be found here: https://github.com/RMorrisGWU/datasets (accessed on 31 December 2022).
